# Advances in systems biology: computational algorithms and applications

**DOI:** 10.1186/1752-0509-6-S3-S1

**Published:** 2012-12-17

**Authors:** Yufei Huang, Zhongming Zhao, Hua Xu, Yu Shyr, Bing Zhang

**Affiliations:** 1 Department of Electrical and Computer Engineering, The University of Texas at San Antonio, San Antonio, TX, 78249, USA; 2 Department of Biomedical Informatics, Vanderbilt University School of Medicine, Nashville, TN 37232, USA; 3 Department of Psychiatry, Vanderbilt University School of Medicine, Nashville, TN 37232, USA; 4 Department of Cancer Biology, Vanderbilt University School of Medicine, Nashville, TN 37232, USA; 5 Center for Quantitative Sciences, Vanderbilt University, Nashville, TN, USA; 6 Department of Biostatistics, Vanderbilt University School of Medicine, Nashville, TN, USA; 7 School of Biomedical Informatics The University of Texas Health Science Center at Houston Houston, TX 77030

## Abstract

The 2012 International Conference on Intelligent Biology and Medicine (ICIBM 2012) was held on April 22-24, 2012 in Nashville, Tennessee, USA. The conference featured six technical sessions, one tutorial session, one workshop, and 3 keynote presentations that covered state-of-the-art research activities in genomics, systems biology, and intelligent computing. In addition to a major emphasis on the next generation sequencing (NGS)-driven informatics, ICIBM 2012 aligned significant interests in systems biology and its applications in medicine. We highlight in this editorial the selected papers from the meeting that address the developments of novel algorithms and applications in systems biology.

## Introduction

The 2012 International Conference on Intelligent Biology and Medicine (ICIBM 2012) was held on April 22-24, 2012 in Nashville, Tennessee, USA [1]. The goal of ICIBM 2012 is to provide a forum to discuss important advancements, exchange ideas, and foster collaborations between leading experts, researchers, and students working in the field computational biology and medicine. The meeting attracted many US-based and international researchers, leading to lively discussions on important research topics, prompting us to consider plans for organizing future ICIBM. Announcements regarding future ICIBM will be available on the conference website [1].

The key components of the scientific program include six technical sessions that cover state-of-the-art research activities in genomics, systems biology, and intelligent computing. The themes of the technical sessions were complemented by a tutorial on proteome informatics and a workshop on next generation sequencing. The conference also featured three keynote presentations by prominent speakers including Dr. Wen-Hsiung Li (University of Chicago), Dr. Randolph A. Miller (Vanderbilt University), and Dr. Brian D. Athey (University of Michigan), who provided valuable discussions on the cutting edge and future developments in the areas of protein structure and function prediction, biomedical computing, and clinical decision support. A more detailed description on the ICIBM 2012 activities is provided in [2]. In addition to a major emphasis on the next generation sequencing (NGS) based informatics, ICIBM 2012 also aligned significant interests in systems biology and its applications in medicine.

Systems biology seeks to understand the function and behavior of complex biological systems through studying the interactions of their components. It usually involves iterative experimental data generation and mathematical modeling, thus requiring a close collaboration among researchers from various disciplines including biology, medicine, computer science, statistics, mathematics, physics and engineering, as demonstrated by the diverse background of the ICIBM 2012 participants. Systems biology has evolved rapidly during the last decade driven by the advancements of high-throughput genomic, transcriptomic, proteomic and metabolomic technologies. It has emerged as an effective framework for using genome-scale data to make predictions, to generate and test hypothesis, and to reveal novel biological insights [3]. Although early works in systems biology focused primarily on model organisms, there is an increasing trend in the application of systems biology in human disease studies [4]. This supplement issue includes 20 articles that describe novel systems biology algorithms and the application of systems biology in biomedical studies. Each article was reviewed by at least two reviewers and went through two rounds of reviews. These articles can be broadly categorized into the following four topics.

### Inference and modeling of gene networks and pathways

Gene networks and pathways are biological systems of genes and proteins that carry out specific biological functions in cell. Difference in gene network topology likely leads to difference in traits or phenotypes of an organism (e.g. different physiological traits in fruit flies, or disease/cancer types and stages). Modeling, reconstruction, and control of gene networks comprise one of the most active areas in systems biology. This supplement issue includes 4 articles on this topic. Qin et al. [5] presents a Bayesian network approach to predict the structure and response of signaling networks by integrating prior biological knowledge in the form of the Ontology Fingerprint. Their approach was applied to the data from the Predictive Signaling Network Modeling Challenge in the fourth Dialog for Reverse Engineering Assessment's and Methods (DREAM4) competition held in 2009. Their approach could accurately capture a signal transduction network of protein kinases and phosphoproteins and was ranked one of the top five best performers in predicting network structure and protein phosphorylation activity under test conditions. Fu et al. [6] also studied the prediction of signal pathways but instead focused on identifying molecular candidates contributing to the priming effect and the corresponding mechanisms. They extended their previous work and proposed an algorithm based on a three-node network model for modeling time-course expression data. The authors applied the algorithm to predict the interferon-γ-mediated priming effect of human macrophages and identified a number of network motifs possibly contributing to interferon-γ priming. Zhang et al. [7] reported a new dynamic time order network model for identifying early and late drug responsive gene targets from time series expression data. The model was applied to expression data of the breast cancer cells treated with estradiol (E2) and successfully identified the time order relations between late response genes of the cell cycle system, suggesting potentials of these genes to serve as biomarkers of E2 treatment. Yang et al. [8] presented a multi-scale differential equation model to predict the left ventricle (LV) remodeling, an important physiological characteristics in cardiac aging. The mode admits disparate measurements including temporal profiles of LV mass, collagen content change, and pressure across LV. The model was applied to real experimental data obtained from over 140 mice and the prediction results captured the major properties of LV remodeling with age.

### Systems approach for disease marker identification

Disease markers are molecular features (DNAs, RNAs, proteins, or metabolites) that can be used for accurate disease diagnosis and prognosis. In contrast to the conventional individual gene based approach, systems approach for marker identification emphasizes the concept of networks, or the connections among molecular features, and aims to identify markers through network analysis or directly identify subnetworks or modules as markers. Five articles in this supplement issue addressed this topic. Shen et al. [9] presented a novel pattern-mining algorithm that detects cancer associated functional subnetworks in human protein-protein interaction (PPI) networks occurring in multiple types of cancer. Validation analysis showed that the discovered subnetworks are functionally enriched and significantly relevant to cancer. Zhu et al. [10] proposed a network based gene prioritization framework to identify and rank candidate genes of orphan disease, or disease that affects a small percentage of the population. The algorithm is based on vertex similarity (VS) and parameter-free. The prediction result was validated on 1598 known orphan disease-causing genes representing 172 orphan diseases and showed that the VS-based approach outperforms Interconnectedness (ICN), a state-of-the-art parameter-free method. Wang et al. [11] investigated the classification of microbiota associated diseases such as pneumonia and dental decay based on bacterial 16S ribosomal RNA profiles. They developed an improved Feature Merging and Selection (FMS) algorithm to identify combinations of 16S rRNA genes that can produce best classification of diseases even when very few microbes are actually shared across communities. Pradhan et al. [12] reported a topological and biological feature-based network approach for identifying gene signatures across colorectal cancer (CRC) populations from gene expression data. Application of this approach to data from four populations identified population-specific clique connectivity profiles (CCPs), which were able to elucidate the divergence among the populations, important biological processes (cell cycle, signal transduction, and cell differentiation), and related gene pathways. Yang et al. [13] reported a core metabolic network of Central Precocious Puberty (CPP), a common pediatric endocrine disease caused by early activation of hypothalamic-pituitary-gonadal (HPG) axis. The network was constructed using the LC-MS measurement of differential urine metabolites in CPP versus normal girls. The result demonstrated that abnormal metabolism of amino acid, especially aromatic amino acid, may have a close correlation with CPP's pathogenesis by activating HPG axis and suppressing hypothalamic pituitary adrenal axis.

### Network and pathway based Genome-wide association studies (GWAS)

Genome-wide association studies (GWAS) have quickly become one of the most important research areas since its first publication in 2005. The major aim is to identify genetic variants that are associated with phenotypic trait or disease under investigation through statistical analyses of several thousands to a few millions of single nucleotide polymorphisms (SNPs) in one experiment. A key problem is that a typical analysis of GWAS data often misses the weak or moderate association signals, as it requires a genome-wide association *P *value (i.e. *P *10^-8^). As a result, so far, the findings from GWAS only represent a small portion of true genetic risk to the disease. Furthermore, the majority of those reported genome-wide associated markers are not functional, making biological interpretation more challenging. This supplement includes 4 articles that employ network- or pathway-based to uncover possible association signals at the systems level and/or pathways aberrantly altered by the genetic variants. Specifically, Han et al. [14] proposed a score-based Bayesian network method, EpiBN, to detect epistatic interactions from GWAS data. EpiBN was tested on simulated datasets and applied to three real disease datasets. Results showed that EpiBN outperforms some other commonly used methods and is especially suitable for detecting epistatic interactions with weak or marginal effects. Liu et al. [15] presented an alternative method that utilizes prior biological knowledge including pathways, network associated with disease, and function of SNPs. The method was applied to a previously published GWAS dataset for type 2 diabetes (T2D) and identified interactions among twelve genes that were not reported in the previous single locus analysis. Interestingly, Hale et al. [16] also investigated T2D SNPs and carried out a network-based analysis. A comprehensive T2D-specific molecular interaction network was constructed, which consists of T2D genetic risk genes and their interacting gene partners. By incorporating T2D risk genes, pathways, and Gene Ontology functional categories, this study revealed that T2D candidate risk genes were located in higher density on chromosome 20. Jia et al. [17] performed an integrative analysis of a GWAS dataset in prostate cancer using pathways and microarray gene expression data. They identified 13 pathways that were aberrantly changed in genetic components and/or at gene expression levels, including the pathways of Fc gamma R-mediated phagocytosis, regulation of actin cytoskeleton, and Jak-STAT signaling pathway. This study demonstrated the feasibility and need to incorporate gene expression data to facilitate pathway analysis of GWAS data.

### Data integration, algorithms, and analysis tools

The remaining papers present new algorithms and analysis tools encompassing diverse aspects of systems biology, including novel network models, feature selection algorithms, visualization and analysis tools for omics data, and evaluation of NGS technologies. Many of them emphasize the need for data integration. It is apparent now that to tackle the enormously complex biological systems, one has to collect and integrate high dimensional data measuring disparate aspects of the systems. Integrating these data in a principled way that conforms to and complements existing biological knowledge is a major challenge in systems biology. To this end, Richards et al. [18] reported a new approach for identifying functionally coherent subsets of genes. The algorithm is based on the framework of graph-spectrum analysis and integrates multiple data sources including the Gene Ontology and co-mentioning of genes in the literature. The algorithm was applied to a real-world dataset and revealed biologically sensible modules. This tool can be used to predict the value of an animal model for drug testing and suggest drug candidates and side effects for disease treatment. Cai et al. [19] proposed a new approach to align neighborhood PPI subnetworks across species. They applied the new algorithm to malaria parasite *Plasmodium falciparum *and *Escherichia coli *and identified 1,082 *Plasmodium falciparum *proteins to be functional orthologous to known transcriptional regulators in the *Escherichia coli *network. The work demonstrated the ability of subnetwork alignment to improve functional annotation of poorly understood organisms. Yu et al. [20] developed a new gene expression profile based cross-species analysis method to analyze similarities in genomic characteristics and drug response between animal models and human diseases. The method was applied to several cases of known animal models and revealed many interesting findings. For example, they found that mouse hypoxia model could accurately mimic the human hypoxia whereas mouse diabetes drug model might have some limitation. Cai et al. [21] presented a new algorithm for detecting protein complexes from affinity purification/mass spectrometry data. The algorithm imposed a bipartite network with one set of nodes for the bait proteins, or known proteins in a complex, and the other set for the prey proteins, or the unknown proteins. An iterative procedure was proposed to predict the prey proteins. Evaluation results showed considerable improvement in prediction accuracy over some well-known algorithms. Su et al. [22] investigated the problem of feature selection in gene expression analysis and discovered flaws in the compound covariate approach proposed in some clinical papers. To correct these flaws, they proposed a random covariate alternative and demonstrated that it could achieve more appropriate results and significantly improve study power for survival outcomes. Jayapandian et al. [23] introduced the Semantic Proteomics Dashboard (SemPoD) platform, which uses provenance together with domain information (semantic provenance) to enable researchers to query, compare, and correlate different types of data across multiple projects, and allow integration with legacy data to support their ongoing research. SemPoD is currently in use at the Case Center for Proteomics and Bioinformatics (CPB). The initial user feedback evaluating the usability and functionality of SemPoD has been very positive and it is being considered for wider deployment beyond the proteomics domain, and in other 'omics' centers. Wang et al. [24] investigated the properties and systematic biases of current NGS technologies in sequencing the genome of *Enterococcus faecium*, a bacterial species that has highly divergent gene contents. The study investigated three different NGS platforms: 454 GS-FLX, Illumina GAIIx, and ABI SOLiD4.0, and showed that each NGS technology displayed some intrinsic properties. They subsequently investigated hybrid assemblies using combinations of different NGS data and reported the parameters that influencing the outcomes of hybrid assemblies. This study implicated the potential guidelines for genome sequencing of microorganisms.

In summary, ICIBM 2012 aligned significant interests in systems biology and its applications in medicine. In addition to novel computational algorithms, works presented in this meeting provide some good examples on the use of systems biology approaches to identify disease genes and pathways, to interpret results from genome-wide association studies, to discover biomarkers for disease diagnosis, prognosis and treatment response, and to find better drug targets. We expect systems biology to continue to play a pivotal role in biological and biomedical research, and it will remain an essential topic in future ICIBM meetings.

## Competing interests

The authors declare that they have no competing interests.
